# Development and Validation of a Gas Chromatography-Mass Spectrometry Method for Determining Acaricides in Bee Pollen

**DOI:** 10.3390/molecules28062497

**Published:** 2023-03-09

**Authors:** Adrián Fuente-Ballesteros, Camille Augé, José Bernal, Ana M. Ares

**Affiliations:** 1 Analytical Chemistry Group (TESEA), I.U. CINQUIMA, Faculty of Sciences, University of Valladolid, 47011 Valladolid, Spain; 2 SIGMA Clermont, Clermont-Ferrand Campus, 63178 Aubiere, France

**Keywords:** acaricides, bee pollen, gas chromatography-mass spectrometry, method validation, QuEChERS, sample treatment

## Abstract

Pesticides can be found in beehives for several reasons, including contamination from surrounding crops or for their use by beekeepers, which poses a risk to bee ecosystems and consumers. Therefore, efficient and sensitive methods are needed for determining pesticide residues in bee products. In this study, a new analytical method has been developed and validated to determine seven acaricides (atrazine, chlorpyrifos, chlorfenvinphos, α-endosulfan, bromopropylate, coumaphos, and τ-fluvalinate) in bee pollen using gas chromatography coupled to mass spectrometry. After an optimization study, the best sample treatment was obtained when using a modified QuEChERS (Quick, Easy, Cheap, Effective, Rugged, and Safe) method employing an ethyl acetate and cyclohexane as the extractant mixture, and a mixture of salts for the clean-up step. A chromatographic analysis (<21 min) was performed in an Agilent DB-5MS column, and it was operated under programmed temperature conditions. The method was fully validated in terms of selectivity, limits of detection (0.2–3.1 µg kg^−1^) and quantification (0.6–9.7 µg kg^−1^), linearity, matrix effect (<20% in all cases), trueness (recoveries between 80% and 108%), and precision. Finally, the proposed method was applied to analyze commercial bee pollen samples, and some of the target pesticides (chlorfenvinphos, α-endosulfan, coumaphos, and τ-fluvalinate) were detected.

## 1. Introduction

Bee pollen is a mixture of flower pollen residues together with nectar or honey, enzymes, wax, and salivary substances from bees, creating small grains [1,2]. It is attracting particular attention as a functional food supplement for human consumption due to its high content of bioactive compounds such as amino acids, phenolic compounds, vitamins, minerals, and lipids [3,4]. This varied composition gives it numerous health promoting effects (antioxidant, anticancer or antimicrobial) [5,6]. However, in recent years, several studies [7,8,9,10] have detected compounds in bee pollen that are harmful to human health, such as pesticides [11], heavy metals and antibiotics. As a result, the image of pollen as a healthy product has been diminished. Pesticides can be found in beehives for several reasons, including contamination from surrounding crops or for its use by beekeepers, posing a risk to bee ecosystems and consumers [10,12,13]. One particular class of pesticides are acaricides, which are used mainly by beekeepers to control *Varroa destructor*. It is an ecto-parasite closely related to spiders and ticks, and lives as an external parasite on bees by feeding on their hemolymph [14]. The worldwide appearance of problems derived from *V. destructor* has led to the adoption of actions for its mitigation, such as the use of acaricides. This situation has led to the application of doses higher than those recommended by legislation [15], which has resulted in the existence of acaricides in beehive products [16], including bee pollen. Therefore, the development of specific methodologies for the determination of acaricides in bee pollen is required.

Bee pollen sample treatments for determining acaricides are problematic because the physico-chemical properties of the analytes make it difficult to remove, interfering with lipids and proteins without losing certain acaricides. Accordingly, it is a significant challenge to develop selective and efficient procedures in this bee product. Acaricides have been studied on bee pollen in recent years using multiple sample treatments, analytical techniques, and detectors. However, they all focused mainly on a modified quick, easy, cheap, effective, rugged, and safe (QuEChERS) method or on solid-phase extraction (SPE)-based sample treatments and the use of chromatography with mass spectrometry (MS) detectors [8,10,17,18]. It should be mentioned that gas chromatography (GC) has been predominantly selected for determining acaricides in bee pollen and other matrices due to the overall physicochemical characteristics of the acaricides [19]. However, some of these, such as coumaphos and chlorpyrifos, have also been determined by means of high-performance liquid chromatography (HPLC) [18].

Thus, the main goal of this paper is to propose an alternative method for the simultaneous determination of seven of the most frequently detected acaricides in bee products around the world (atrazine, chlorpyrifos, chlorfenvinphos, α-endosulfan, bromopropylate, coumaphos, and τ-fluvalinate; see Table 1) in bee pollen samples through the use of GC-MS. We have adapted the GC-MS conditions from a recent study of our group [19], as we wanted to focus our efforts in developing an efficient, simple, economic, and fast sample treatment. In addition, we have performed the method optimization with the aim of obtaining the best extraction efficiency (recovery values), minimizing the matrix effect as much as possible, and fulfilling the principles of green analytical chemistry (reducing the number and amount of reagents, reducing time, and costs) [20]. Another of our objectives was to validate the method according to the current legislation [21], and to apply it to the analysis of multifloral bee pollen samples from different origins (commercial and from experimental apiaries).

## 2. Results and Discussion

### 2.1. Optimization of Sample Treatment

It is a well-known fact that the origin of bee pollen (geographical and botanical) is a main characteristic that significantly influences the analytical determination and chromatographic profile; even bee pollen of the same botanical origin can exhibit differences in their composition [22]. For these reasons, we decided to start by testing bee pollen samples in SCAN and SIM (Selected Ion Monitoring) mode (data not shown) (*n* = 8; S1–S8) of different geographical origins purchased in local supermarkets, and using a similar procedure to that employed for determining acaricides in light honeys [19]. The SCAN mode provided a global visualization of the compounds present in the matrix.

For two of the bee pollen samples we found the presence of two interfering signals from matrix components that affected chlorpyrifos and α-endosulfan peaks. Regarding the other samples, we detected only one interference with the chlorpyrifos peak. The main differences between the peaks were derived from their intensity and were due to the presence of other smaller interferences. The problem of co-elution with the analytes peaks was subsequently solved with the remaining optimization steps. Next, we moved to optimize the bee pollen sample amount. In the scientific literature there are some articles where the amount of bee pollen sample employed is high, i.e., 15 g [17] or 5 g [9,23,24]. Our objective was to reduce the amount of sample for reducing the matrix effect, but without affecting other parameters such as extraction efficiency or sensitivity. Therefore, we performed several tests by using 1 and 2 g of bee pollen sample. Spiked samples were injected and chromatograms were analyzed. We found that the amount of sample used did not significantly influence the number of extracted compounds, and the intensity of the obtained peaks was not different, therefore 2 g of sample were chosen for continuing with the optimization study.

Subsequently, we tackled the optimization of the extractant solvent. Different solvents haven been used to extract pesticides, including acaricides, from bee pollen, with acetonitrile [8,10,17,18,23,25,26], hexane [17,25,27], and acetic acid normally being employed [18]. The current trend in sample treatment prioritizes the use of solvents that are as least toxic as possible, in addition to being environmentally friendly [28], such as ethyl acetate and cyclohexane, which were evaluated in the presented study. Moreover, a combination of these solvents has been previously employed in the sample treatment of beehive products when determining pesticides [12,19,29]. Therefore, we decided to check the suitability of five different solvent mixtures that were chosen according to the related literature and previous experiments (see Supplementary Materials, Table S1). It should be mentioned that the optimization process began with our decision to use 2 g of bee pollen, which was mixed with 3 mL of ultrapure water in a centrifuge tube. After 30 s of shaking time, 10 mL of the extractant mixture was added, and the resulting mixture was shaken for 2 min. Next, magnesium sulfate (1.4 g) and sodium chloride (0.4 g) were added, and the mixture was shaken again for 1 min (vortex). The mixture was then centrifuged (5 min, 5 °C, 7500 rpm), and 5 mL of supernatant were collected and evaporated to dryness at 30 °C. Finally, the dry residue was reconstituted with 1 mL of the extraction mixture (for samples spiked before sample treatment; BF samples) or 1 mL of the internal standard (IS, chlorfenvinphos-d_10_) at 0.1 mg L^−1^ (for samples spiked after sample treatment; AF samples). It was observed that the peak areas were much higher in the mixture in the ethyl acetate and cyclohexane mixture, obtaining better recovery values, and a lower matrix effect. Regarding the mixture ratios, different combinations (20:80, 50:50; *v*/*v*) were tested, and the results showed that the best results (extraction efficiency and matrix effect) were obtained in all cases when employing the 50:50 (*v*/*v*) mixture. Next, the influence of the extractant volume (5, 10 and 15 mL) and shaking time (1, 2 and 5 min) on extraction efficiency was studied. The recovery values were good enough (>80%; data not shown) and quite similar for the two options that used either the largest volumes (10 and 15 mL), or the longest shaking times (2 and 5 min), and consequently it was decided to select 10 mL and 2 min to reduce the solvent consumption and procedure time.

It should be noted that the matrix effect and recovery could not be calculated for chlorpyrifos at this stage due to the presence of a matrix interfering signal. Therefore, the next goal of the optimization procedure was to try to remove of this interference. Some tests were performed using the QuEChERS dSPE EMR-Lipid kit, which has provided good results in previous works [30], but in this case, it was not possible to remove the interference. It was also considered to include an additional freezing step to the protocol for 15 min before the dispersive-SPE (dSPE) step. The results indicated that the removal of the interfering signal was achieved, but the extraction efficiency and the matrix effect influence were worse for all of the compounds, particularly for atrazine (from 98 to 57% in the recovery values) and coumaphos (from −4 to −34 in relation to matrix effect). For this reason, the freezing step was discarded. We then decided to evaluate different options for the dSPE step. This mainly involved the use of primary secondary amine (PSA) to remove organic acids and polar pigments, C_18_ to eliminate some lipids, and magnesium sulfate to remove the remaining water. Our first test was performed by taking 2 mL of supernatant and mixing it with a mixture of PSA (0.5 g), C_18_ (0.5 g), and magnesium sulfate (1.5 g) to remove the interference that affected the determination of chlorpyrifos. The results demonstrated the suitability of these conditions, as the interference was removed, while a good extraction efficiency (80–108%) was also observed, as was the absence of a significant matrix effect (<20%) for all compounds (see Table 2). To further optimize the protocol and make it more economical, different amounts/combinations of the above-mentioned salts were evaluated (PSA, 0.05–0.50 g; C_18_, 0.10–0.50; MgSO_4_, 0.5–1.5 g). However, the results were not adequate because the number of interference peaks increased, and the matrix effect did not significantly improve (data not shown). Therefore, we decided to maintain the quantities of the salts that were initially selected.

To sum up, we have proposed a sample preparation method that can be considered as a promising alternative to the existing procedures since it is fast (<21 min), simple, efficient, and environmentally friendly due to the nature of the solvents selected to perform the extraction of the analytes (ethyl acetate and cyclohexane). On top of that, the recoveries were satisfactory for all the acaricides in the bee pollen (80–108%; see Table 2) and, most importantly, it was determined that the matrix effect was not significant in any of them.

### 2.2. Method Validation

The method validation was based on current legislation for pesticides residues analysis in food [21] and recent publications of our group [12,19]. Validation was performed with blank bee pollen, standards in the solvent, and standards in matrix extracts obtained according to the selected sample treatment. The specific procedures for determining the different validation parameters are summarized in the following sections.

#### 2.2.1. Selectivity

**Figure 1:**
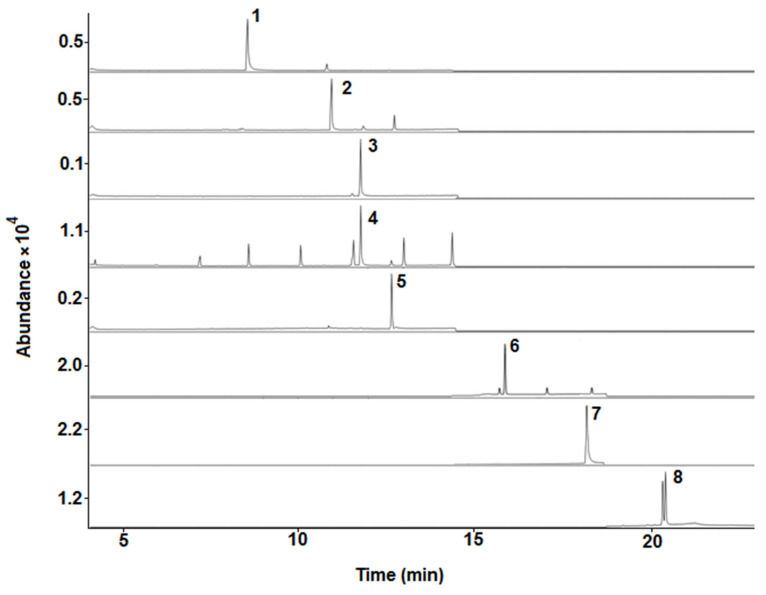
Representative GC-MS chromatograms (SIM mode using the quantification/target ions; see Table 1) obtained from a standard in solvent mixture (0.5 mg L^−1^; IS, 0.1 mg L^−1^). **1**, atrazine; **2**, chlorpyrifos; **3**, chlorfenvinphos-d_10_ (IS); **4**, chlorfenvinphos; **5**, α-endosulfan; **6**, bromopropylate; **7**, coumaphos; **8**, τ-fluvalinate.

**Figure 2:**
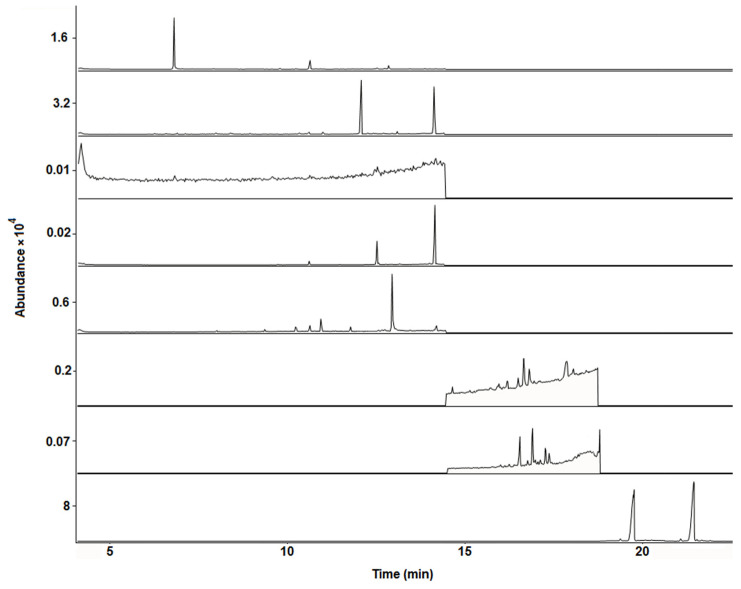
Representative GC-MS chromatograms (SIM mode using the quantification/target ions; see Table 1) obtained from a blank multifloral bee pollen sample free of acaricides (E1).

Selectivity was evaluated by comparing the chromatograms and mass spectra of standards in solvents, matrix-matched standards, and blank bee pollen samples. No chromatographic interferences of matrix compounds were observed at analytes retention times when comparing blank bee pollen samples and standards in solvents (see Figure 1 and Figure 2). Moreover, a significant similarity between the mass spectrum of the acaricides under study in the solvents and standards in matrix extracts was observed (see Supplementary Materials, Figure S1).

The relative intensities of the selected ions for each acaricide in both types of standards were compared and, for all cases, they were within ±15% of the relative intensity (data not shown), which is lower than the maximum values allowed (±30%) [21].

#### 2.2.2. Limits of Detection and Quantification

The limits of detection (LODs) and quantification (LOQs) were determined by the injection of several blank samples’ measurement noise at the elution times for the analytes, and comparing this response (mean values) with the signal (peak heights) of acaricides at low concentration levels. LODs and LOQs were estimated to be three and ten times the signal/noise ratio. LODs-LOQs values ranged from 0.2 to 3.1 µg kg^−1^ and from 0.6 to 9.7 µg kg^−1^, respectively (see Table 3), and were below the maximum residue levels (MRLs) established by legislation [15], showing an excellent sensitivity of the prosed analytical method. Those values are comparable to the best values obtained in previous publications [8,27].

#### 2.2.3. Matrix Effect

To ascertain the matrix correlated ESI ionization for the acaricides, a comparison was made of the detector responses by comparing the analyte peak with standard in solvent (matrix-free) solutions and AF samples at the three different concentration levels (low, medium, and high). The parameter was calculated with the following equation: 100 × [1 − (peak area of analyte in AF sample/peak area of IS in BF sample)/(peak area of analyte in standard in solvent/peak area of IS in standard in solvent)]. The analyte responses at the three QC levels assayed ranged from 2% to 15% (see Table 2), which implies that the matrix did not affect acaricide signals, and in all cases comprised ±20% of signal suppression or enhancement. In addition, the slope confidence intervals with standards in solvent and standards in matrix extracts were also compared, finding that they overlapped in all cases (see Table 3). No statistical differences were found; therefore, it can be concluded that the matrix effect did not affect analyte ionization, which posed a noteworthy advantage of the proposed sample treatment compared with other previous proposals [8,18,27].

#### 2.2.4. Working Range

Standard solvent calibration curves were used to quantify the acaricides in bee pollen. The concentration of the analytical curves varied between LOQ and 1000 μg L^−1^ (LOQ, 50, 100, 250, 500, and 1000 μg L^−1^), which corresponds to concentrations between LOQ and 700 μg kg^−1^ (LOQ, 100, and 700 μg kg^−1^). Calibration curves (see Section 3.2) were constructed using a linear fitting, and not forced to zero by plotting the analyte concentration on the *x*-axis against the analyte peak area/IS area on the *y*-axis. Linearity was evaluated by visual analysis of the plots; a calculation was made of the determination coefficients (R^2^), and by our back calculation of the concentration of the individual calibration standards. The graphs obtained in the calibration curves were straight lines, with R^2^ values higher than 0.99 in all cases (see Table 3). The deviation of back-calculation concentration from true concentration was lower than 20% (data not shown), as specified by European legislation [21]. Standard solvent calibration curves were used to quantify the acaricides in bee pollen samples because of the absence of the matrix effect.

#### 2.2.5. Precision

Precision was expressed as relative standard deviation (%RSD) and was performed concurrently by repeated sample analysis using BF samples at three different concentration levels (low, medium, and high), either on the same day (intraday precision) [21], or over three consecutive days (interday precision) [21]. Values were lower than 9% in all cases (see Supplementary Materials, Table S2), which is consistent with the current European legislation (%RSD ≤ 20%) [21], and similar to or better than the precision values reported in previous methods [8,17,18,27].

#### 2.2.6. Trueness

Trueness was evaluated by means of recovery experiments (as a measure of trueness), by comparing the results (analyte peak area/IS area) obtained from blank bee pollen samples spiked at three different concentrations (low, medium, and high levels), either prior to or following sample treatment. Mean recoveries ranged from 80% to 108%, with %RSD values lower than 9% in all cases (see Table 2). Those values fulfilled the requirements established by the European legislation (recovery percentages between 70% and 120%; RSD ≤ 20%) [21].

### 2.3. Application of the Method

The proposed and validated method was applied for determining potential acaricide residues in 12 bee pollen samples from local supermarkets (*n* = 8; S1–S8) and obtained from experimental apiaries (*n* = 4; E1–E4). Analyses were performed in triplicate and IS was added to all samples. Six of the pollen samples analyzed (S1–S3, S5, S6, and E3) had one or more of the compounds studied, and in most cases in concentrations higher than those authorized by current legislation (see Table 4).

Bee pollen samples S1–S3, S5, S6 and E3 presented acaricide concentrations above the established MRLs (see chromatogram of E3 sample in the Supplementary Materials, Figure S2). By contrast, τ-fluvalinate and coumaphos levels in S3 and S6 bee pollen samples were below these values. S4, S7, S8, E1, E2 and E4 bee pollen samples were free of acaricides, or at least below to LOD. Out of seven acaricides, four (chlorfenvinphos, coumaphos, α-endosulfan, and τ-fluvalinate) are present in the bee pollen samples, with τ-fluvalinate exhibiting the highest pesticide concentration [10,31]. Moreover, it can be observed that the F sample presents three out of seven pesticides and that the acaricide content is higher in commercial pollen than samples obtained from experimental apiaries. Similarly, these results are in line with the findings found in other beehive products such as honey [19], where a high persistence of τ-fluvalinate was identified, and its levels in honey did not decrease after eight months in the dark at 35 °C [32].

The presence of acaricide in high concentrations compared to the MRLs is a matter of great concern for bees, as well as consumers. In fact, bees suffer from episodes of poisoning, altered flight ability, poor sperm viability, larval survival, and altered gene expression due to the use of pesticides. Indirect pesticide applications can pose a risk to the ecosystem and to bees and consumers. In fact, this could ultimately lead to an even greater loss of these pollinating insects if any type of pesticide continues to be widely used, which would have serious consequences for the supply of bee products.

## 3. Materials and Methods

### 3.1. Reagents and Materials

Acaricide standards (atrazine, chlorpyrifos, chlorfenvinphos, α-endosulfan, bromopropylate, coumaphos, τ-fluvalinate, and chlorfenvinphos-d_10_; see structures in Table S3; Supplementary Materials), all of analytical-grade and with purity greater than 99%, were purchased from Dr. Ehrenstorfer (Augsburg, Germany).

All solvents (ethyl acetate, cyclohexane, hexane, acetonitrile, triethylamine, and acetic acid) were of chromatographic grade and were obtained from VWR Prolabo Chemicals (Fontenay-sous-Bois, France). Ultrapure water was obtained using a Millipore Milli-RO plus and Milli-Q systems (Bedford, MA, USA). A vortex mechanical mixer from Heidolph (Schwabach, Germany), a thermostated ultrasound bath, a drying oven, and a vibromatic mechanical shaker were all supplied by J.P. Selecta S.A. (Barcelona, Spain); a 5810 R refrigerated bench-top centrifuge from Eppendorf (Hamburg, Germany), a R-3 rotary evaporator from BUCHI (Flawil, Switzerland), and Nylon syringe filters (17 mm, 0.45 μm; Nalgene, Rochester, NY, USA) were employed for sample treatment. In addition, QuEChERS dSPE enhanced matrix removal lipid (EMR-Lipid) sorbent was supplied by Agilent Technologies (Folsom, CA, USA). For the clean-up step, magnesium sulfate was obtained from Sigma-Aldrich Chemie Gbmh (Steinheim, Germany), sodium chloride was supplied by Panreac (Barcelona, Spain), while PSA and C_18_ were provided by Supelco (Bellefonte, PA, USA).

### 3.2. Standards

Standard stock (≈1000 mg L^−1^) and working solutions of the studied acaricides were prepared in a mixture of ethyl acetate and cyclohexane (50:50, *v*/*v*). Bee pollen samples (2.0 g), in which the absence of acaricide residues had been previously confirmed using GC-MS (blank samples) were spiked with variable amounts of the analytes before (BF samples) or after (AF samples) sample treatment to prepare the standard in matrix extracts. The spiking of the samples was done similarly to Ares et al. [3]. Briefly, representative portions of the blank bee pollen were weighed and transferred to a crystallizer, where they were homogeneously spiked with working standard solutions. The mixtures were stirred with a glass rod to assist the homogenization and left to equilibrate overnight prior to analysis. Meanwhile, AF samples were prepared by spiking blank pollen samples, which were previously treated with the proposed sample treatment, with working standard solutions that were added to the elution solvent. The internal standard (IS; chlorfenvinphos-d_10_) was always added at the same concentration (0.1 mg L^−1^).

These samples were used for validation (spiked samples (low, medium, and high) and calibration curves), and sample treatment studies. It must be noted that three replicates, which were injected three times, were prepared for all of the studies. Each spiked sample was prepared with 2 g of blank bee pollen samples spiked with three different concentrations of the acaricides within the linear range. These were as follows: low-LOQ (see Table 3); medium-100 µg kg^−1^; high-700 µg kg^−1^. It is worthy of note that the recovery percentages of each single acaricide from bee pollen at the three different concentration levels are summarized in Table 2. The standard stock solutions were stored in glass containers in darkness at −20 °C; working and standard matrix solutions were stored in glass containers and kept in the dark at +4 °C.

### 3.3. Sample Procurement and Treatment

#### 3.3.1. Samples

Multifloral bee pollen samples (*n* = 12) were kindly donated by the Center for Agroenvironmetal and Apicultural Investigation (Marchamalo, Guadalajara, Spain) or purchased in local markets (Valladolid, Spain). To homogenize each of these samples, they were dried at 45 °C in an oven, individually ground in a mill, and pooled for optimum sample homogeneity, and subsequently stored in darkness at 4 °C until analysis. Three replicates (sub-samples) of each sample, which were injected in triplicate, were examined to determine the acaricide content.

#### 3.3.2. Sample Treatment

**Figure 3:**
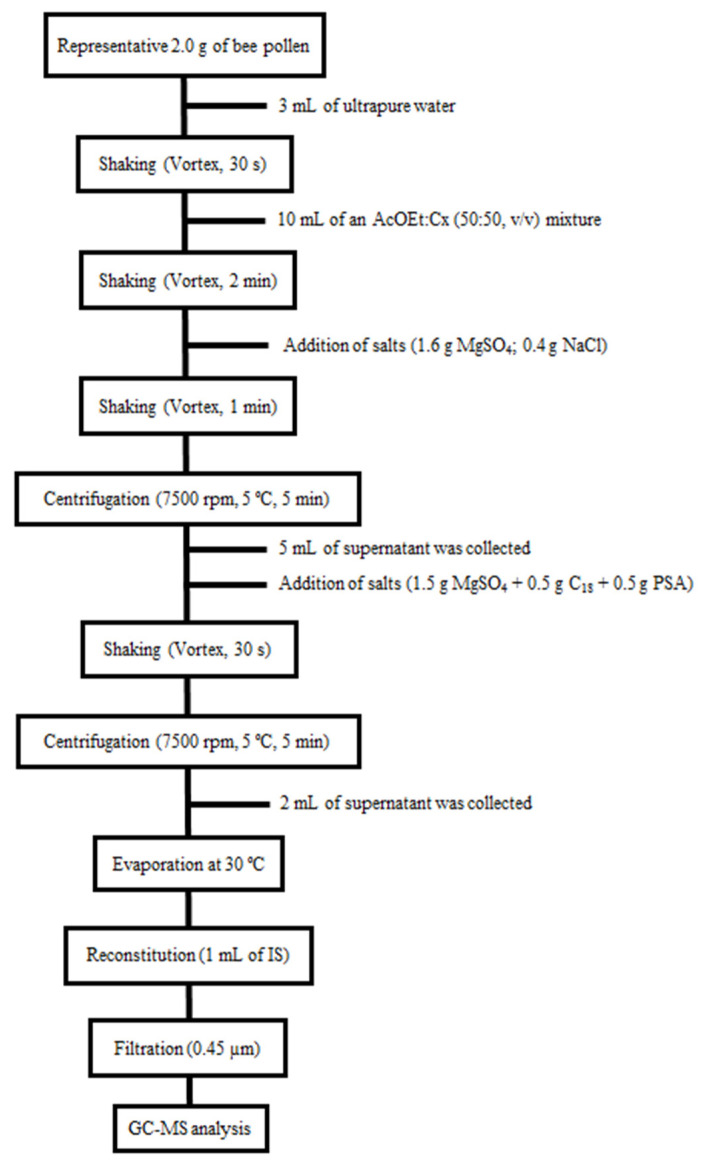
Analytical procedure work-up flow chart for bee pollen sample preparation.

Briefly, a representative amount of bee pollen was ground to fine powder. An amount of 2.0 g of homogenized of sample was weighed in a 50 mL centrifuge tube, after which 3 mL of ultrapure water was added, and the tube was shaken for 30 s in a vortex device (2500 rpm). Next, 10 mL of an ethyl acetate and cyclohexane (50:30, *v*/*v*) mixture was added to the tube and then shaken again in a vortex device for 2 min. After that, a mixture of salts (1.6 g magnesium sulfate: 0.4 g sodium chloride) was added and shaken in vortex for 1 min (2500 rpm). The resulting mixture was centrifugated (7500 rpm, 5 °C) for 5 min and, subsequently, 5 mL of the supernatant was collected and placed on a salt mixture (1.5 g magnesium sulfate, 0.5 g C_18_, 0.5 g PSA). The tube was then shaken in a vortex device for 30 s (2500 rpm). A centrifugation step (7500 rpm, 5 °C) was again performed for 5min, and 2 mL of the extract was collected and evaporated to dryness at 30 °C in a rotary evaporator. Finally, the dry extract was reconstituted with 1 mL of an IS solution (0.1 mg L^−1^), and it was passed through a 0.45 μm nylon filter prior to GC-MS analysis. Figure 3 outlines the steps of the procedures used during the present study.

### 3.4. GC-MS Conditions

An Agilent Technologies (Palo Alto, CA, USA) 7890A GC coupled to an Agilent Technologies 5975C MS equipped with an ALS 7693B autosampler and a MS ChemStation E 01.00.237 software (Agilent Technologies) was used. The chromatographic column was an Agilent DB-5MS (30 m × 0.25 mm × 0.2 μm). The GC-MS parameters were selected according to previous work [19]. The GC was operated under programmed temperature conditions, from 60 °C (1 min) to 170 °C (5 min), at 40 °C/min and then increased to 310 °C (3 min) at 8 °C/min. An injection volume of 1 µL was employed with the autosampler in pulsed splitless mode, the injector temperature set at 280 °C, and helium (Carburos Metálicos, Barcelona, Spain) was used as the carrier gas at a flow-rate of 1.2 mL/min. MS SCAN parameters included a mass range of 50–400 m/z, operating in electron ionization mode with an ionization energy of 70 eV. The ion source and quadrupole temperatures were 230 °C and 150 °C, respectively. Analyses were performed in SIM mode, with one target/quantification and two qualifier ions for each analyte (see Table 1). Under optimal GC-MS conditions, all compounds eluted in less than 21 min (see Figure 1). It should be highlighted that τ-fluvalinate showed two chromatographic peaks, which is because this compound presents a diastereomeric pair of compounds [19,33]. τ-Fluvalinate contains two chiral centers, and commercial formulations of this compound have one center, next to the amino group (see Supplementary Materials, Table S3), fixed in the R configuration. Therefore, the R, S configuration at the other chiral center (next to the cyano group) leads to a diastereomeric pair of compounds with different properties [33]. Thus, the sum of their corresponding areas was employed for quantification purposes. In addition, the three separated signals observed close to the bromopropylate peak could be related to impurities or degradation products [34].

## 4. Conclusions

In this work, a new analytical method was developed and validated to determine seven acaricides (atrazine, chlorpyrifos, chlorfenvinphos, α-endosulfan, bromopropylate, coumaphos, and τ-fluvalinate) in bee pollen samples using a modified QuEChERS method (an ethyl acetate and cyclohexane as extractant and a mixture of salts for clean-up step) and gas chromatography-mass spectrometry. After a sample treatment optimization process, the one that provided the best performance (extraction efficiency and matrix effect) was selected according to simplicity, cost, and time-consumption. The recoveries obtained were sufficiently good, and the matrix effect was avoided for all of the acaricides, which represents an advantage compared to most of the existing methods in the scientific literature. The chromatographic conditions were adapted from previous works of the group, achieving the chromatographic separation of the acaricides in less than 21 min. The proposed method was fully validated according to current legislation. The obtained results showed that the analytical performance of the method was similar or better than previous proposals. The LODs and LOQs obtained were lower than the MRLs established for the compounds studied in bee pollen, and were comparable with the best published values. Finally, the validated method was applied to analyzed commercial bee pollen, and samples were obtained from experimental apiaries. Some of the studied pesticides (chlorfenvinphos, α-endosulfan, coumaphos, and τ-fluvalinate) were detected, and in some cases at concentrations higher than those authorized by legislation.

## Figures and Tables

**Table 1:** GC-MS data.

Acaricide	Family	Retention Time (min)	Target Ions (*m/z*)	Qualifier Ions (*m/z*)
Atrazine	Triazines	8.5	200	173, 215
Chlorpyrifos	Benzylates	10.8	197	258, 314
Chlorfenvinphos	Organophosphates	11.8	267	270, 329
α-Endosulfan	Organophosphates	12.6	241	195, 207
Bromopropylate	Organophosphates	15.9	341	183, 185
Coumaphos	Organophosphates	18.2	362	109, 226
τ-Fluvalinate	Pyrethroids	20.4	250	181, 208
Chlorfenvinphos-d_10_	-	11.7	333	-

**Table 2:** Evaluation of the extraction efficiency (recoveries) and the matrix effect of the sample treatment (mean ± %RSD; three replicates that were injected in triplicate).

Acaricide	Evaluation of the Extraction Efficiency			Evaluation of the Matrix Effect		
	Mean (%) ± RSD (%)			Mean (%) ± RSD (%)		
	Low Level	Medium Level	High Level	Low Level	Medium Level	High Level
Atrazine	80 ± 2	84 ± 2	82 ± 5	2 ± 2	−2 ± 5	3 ± 3
Chlorpyrifos	102 ± 6	108 ± 3	101 ± 6	−12 ± 3	−8 ± 5	−7 ± 6
Chlorfenvinphos	89 ± 4	88 ± 5	86 ± 3	−8 ± 1	−2 ± 2	−4 ± 2
α-Endosulfan	84 ± 5	88 ± 5	81 ± 2	−18 ± 5	−17 ± 3	−12 ± 4
Bromopropylate	91 ± 2	102 ± 2	95 ± 5	4 ± 6	6 ± 4	3 ± 3
Coumaphos	102 ± 4	96 ± 4	97 ± 3	6 ± 5	13 ± 6	10 ± 3
τ-Fluvalinate	100 ± 4	107 ± 6	103 ± 4	10 ± 2	15 ± 2	14 ± 2

Low level, LOQs (see Table 3); Medium level, 100 µg kg^−1^; High level, 700 µg kg^−1^; RSD, relative standard deviation.

**Table 3:** Calibration curve data, LOD, LOQ, and MRL values.

Compound	Standards in Solvent		Standards in Matrix		LOD (µg kg^−1^)	LOQ (µg kg^−1^)	MRL (µg kg^−1^)
	SCI	R^2^	SCI	R^2^			
Atrazine	30.1 ± 2.2	0.999	30.6 ± 2.1	0.998	3.1	9.7	50
Chlorpyrifos	21.7 ± 3.1	0.998	19.7 ± 1.9	0.999	2.4	8.5	10
Chlorfenvinphos	35.1 ± 4.1	0.998	33.4 ± 2.6	0.999	1.1	3.7	10
α-Endosulfan	6.5 ± 3.3	0.998	5.5 ± 3.5	0.998	0.2	0.6	10
Bromopropylate	52.9 ± 1.3	0.996	55.1 ± 1.8	0.999	0.8	2.8	10
Coumaphos	11.5 ± 2.2	0.998	12.8 ± 2.7	0.999	1.2	4.1	100
τ-Fluvalinate	75.3 ± 3.7	0.998	84.6 ± 3.3	0.999	2.8	9.3	50

SCI, slope confident intervals; LOD, limit of detection; LOQ, limit of quantification; R^2^, determination coefficient; MRL, maximum residue limit.

**Table 4:** Results (means of triplicate analyses (µg kg^−1^); %RSD < 9% in all cases) of the investigation of acaricides in bee pollen samples from different origins. The other acaricides under study were below LOD in the samples.

Sample	Chlorfenvinphos	α-Endosulfan	Coumaphos	τ-Fluvalinate
S1	35	<LOD	<LOD	<LOD
S2	<LOD	<LOD	<LOQ	24
S3	30	<LOQ	<LOQ	10
S4	<LOD	<LOD	<LOD	<LOD
S5	<LOQ	<LOD	<LOD	31
S6	32	77	42	<LOD
S7	<LOD	<LOD	<LOD	<LOD
S8	<LOD	<LOD	<LOD	<LOD
E1	<LOD	<LOD	<LOD	<LOD
E2	<LOD	<LOD	<LOD	<LOD
E3	<LOD	<LOD	<LOD	97
E4	<LOD	<LOD	<LOD	<LOD

<LOD, below limit of detection; <LOQ, below limit of quantification.

## Data Availability

The datasets generated during the current study are contained within this article and the Supplementary Materials, or they are available from the corresponding author on reasonable request.

## Supplementary Materials

The following supporting information can be downloaded at: https://www.mdpi.com/article/10.3390/molecules28062497/s1, Figure S1: MS spectra of chlorfenvinphos in standard in (A) solvent and (B) in matrix at the same concentration (0.5 mg L^−1^); Figure S2. Representative GC-MS chromatogram (SIM mode using the quantification/target ions; see Table 1) obtained from multifloral bee pollen sample (E3; 97 µg kg^−1^) with endogenous τ-fluvalinate content over LOQ. The GC-MS conditions are summarized in Section 3.4 and Table 1; Table S1: Evaluation of the extraction efficiency (recovery percentages) and the matrix effect when employing 10 mL of different solvent mixtures with spiked blank bee pollen samples at medium level (100 µg kg^−1^) (mean ± %RSD; three replicates that were injected in triplicate); Table S2: Summary of precision studies (minimum and maximum %RSD values) for the determination of acaricides in spiked blank bee pollen samples; Table S3: Chemical structure of the studied acaricides.

## Sample Availability

Some of the bee pollen samples are available from the authors on reasonable request.
