# Supplementary material for: Development and Validation of a Gas Chromatography-Mass Spectrometry Method for Determining Acaricides in Bee Pollen
Source: Molecules. 2023 Mar 9;28(6):2497. doi: 10.3390/molecules28062497 (PMC10056623; doi:10.3390/molecules28062497)

SUPPLEMENTARY MATERIALS

Development and Validation of a Gas Chromatography-Mass Spectrometry Method for Determining Acaricides in Bee Pollen

Adrián Fuente-Ballesteros[1], Camille Augé[2], José Bernal[1], and Ana M. Ares[1,*]

[1] Analytical Chemistry Group (TESEA), I.U. CINQUIMA, Faculty of Sciences, University of Valladolid, 47011 Valladolid, Spain;
[2] SIGMA Clermont, Clermont-Ferrand Campus, 63178 Aubiere, France
* Correspondence: ana.maria.ares@uva.es; Tel.: +34-983184249

Table S1. Evaluation of the extraction efficiency (recovery percentages) and the matrix effect when employing 10 mL of different solvent mixtures with spiked blank bee pollen samples at medium level (100 µg kg^{-1}) (mean ± %RSD; three replicates that were injected in triplicate).

Acaricide	ACN/AcOH (99:1 ; v/v)		ACN/TEA (98:2 ; v/v)		ACN/Hx (50:50 ; v/v)		AcOEt/Cx (20:80 ; v/v)		AcOEt/Cx (50:50 ; v/v)	
	**EE**	**ME**	**EE**	**ME**	**EE**	**ME**	**EE**	**ME**	**EE**	**ME**
**Atrazine**	10	16	111	48	98	1	80	-2	85	-2
**Chlorpyrifos**		-	51	-21	35	-32	-	-	-	-
**Chlorfenvinphos**	11	3	56	-2	64	-11	99	-10	108	-8
**α-Endosulfan**	7	-4	53	-29	33	-28	89	-30	90	-15
**Bromopropylate**	15	5	54	50	35	31	85	15	104	7
**Coumaphos**	12	15	86	77	44	83	92	20	98	10
**τ-Fluvalinate**	15	1	38	43	45	-13	101	14	109	16

ACN, acetonitrile; **AcOEt**, ethyl acetate; **AcOH**, acetic acid; **Cx**, cyclohexane; **EE**, extraction efficiency; **Hx**, hexane; **ME**, matrix effect; **TEA**, tryethylamine.

Table S2. Summary of precision studies (minimum and maximum %RSD values) for the determination of acaricides in spiked blank bee pollen samples.

	**Spiking Level**	**Multifloral Bee Pollen**	
		Min.	Max.
**Intraday precision**	Low	2	5
	Medium	4	8
	High	3	7
**Interday precision**	Low	4	7
	Medium	4	7
	High	2	5

Low level, LOQ, (see **Table 3**); **Medium level**, 100 μg kg^{-1}; **High level**, 700 μg kg^{-1}.

Table S3. Chemical structure of the studied acaricides.

**Atrazine**	**Chlorpyrifos**
CAS number: 1912-24-9	CAS number: 2921-88-2
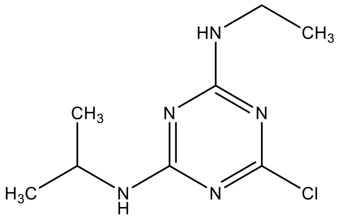	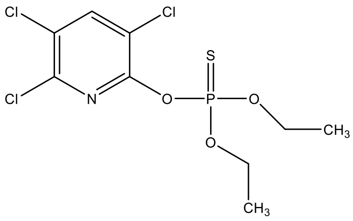
**α-Endosulfan**	**τ-Fluvalinate**
CAS number: 959-98-8	CAS number: 102851-06-9
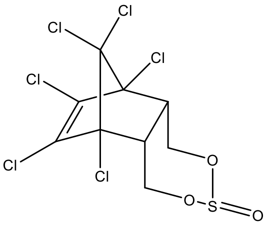	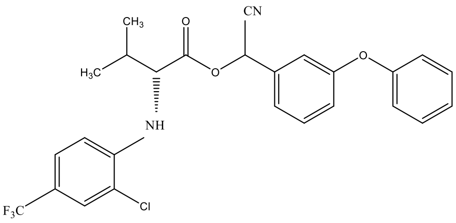
**Chlorfenvinphos**	**Chlorfenvinphos-d10**
CAS number: 470-90-6	CAS number: 1346606-54-9
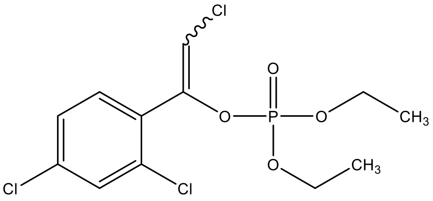	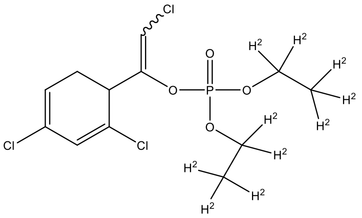
**Bromopropylate**	**Coumaphos**
CAS number: 18181-80-1	CAS number: 56-72-4
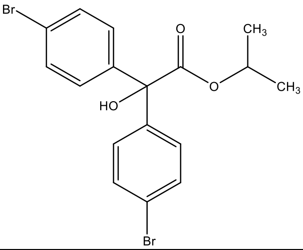	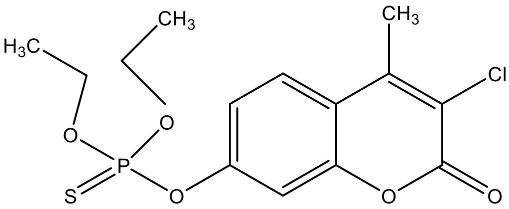

Figure S1. MS spectra of chlorfenvinphos in standard in (A) solvent and (B) in matrix at the same concentration (0.5 mg L^{-1}). The GC-MS conditions are summarized in subsection 3.4 and **Table 1**.

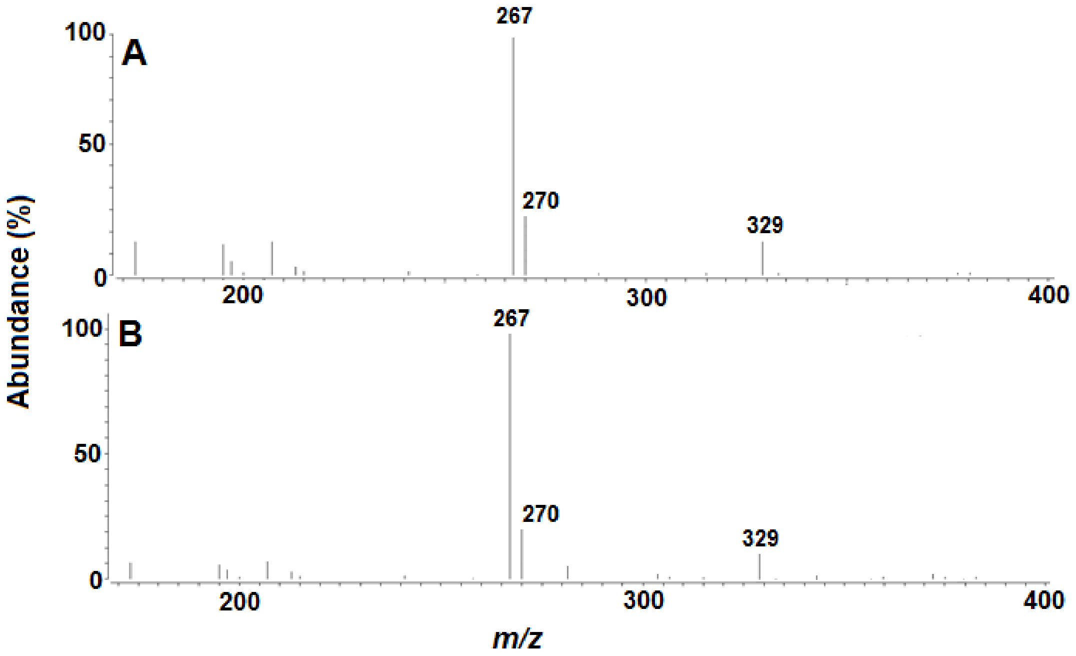

Figure S2. Representative GC-MS chromatogram (SIM mode using the quantification/target ions; see **Table 1**) obtained from multifloral bee pollen sample (E3; 97 µg kg^{-1}) with endogenous τ-fluvalinate content over LOQ. GC-MS conditions are summarized in Subsection 3.4 and **Table 1.**

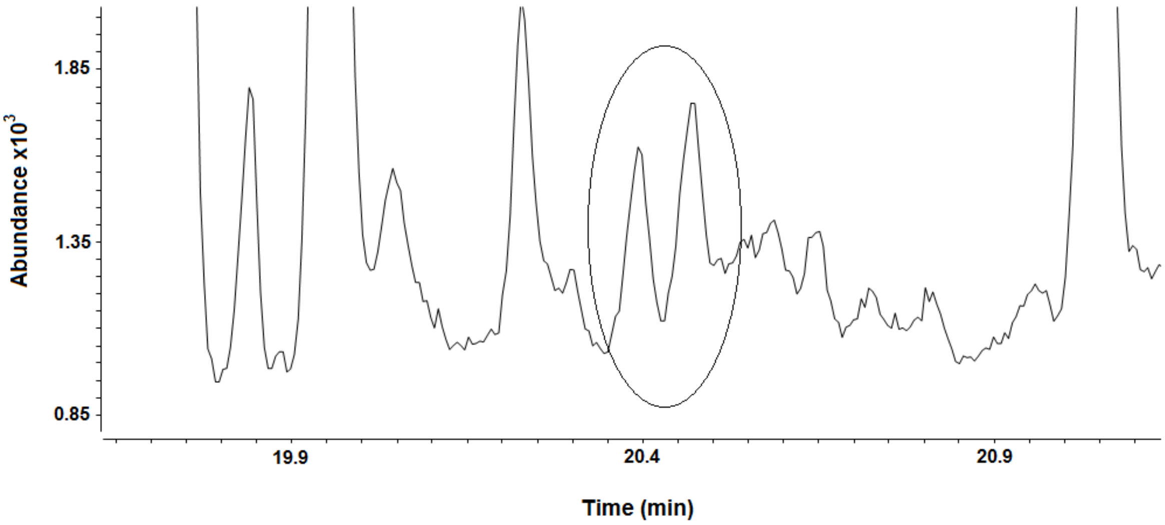

Supplement: Supplementary file 1 [file molecules-28-02497-s001.zip › molecules-2266034-supplementary.pdf]
